# Mary Cassatt: Impressionism, Tibial Torsion, Metatarsus Adductus, and Asymmetric Thigh Skin Folds

**DOI:** 10.7759/cureus.105782

**Published:** 2026-03-24

**Authors:** James G Gamble

**Affiliations:** 1 Department of Orthopaedic Surgery, Division of Pediatric Orthopaedics, Stanford University School of Medicine, Palo Alto, USA

**Keywords:** asymmetric skin fold, developmental dysplasia of the hip, gait abnormality, metatarsus adductus, tibial torsion

## Abstract

Mary Cassatt was one of the three great female impressionists along with Berthe Morisot and Marie Bracquemond. Most of Cassatt's paintings focused on domestic scenes of mothers, infants, and children, almost exclusively female. Her paintings depicted children with common pediatric orthopaedic conditions like tibial torsion, metatarsus adductus, and asymmetric medial thigh skin folds. It was during this same time that clinical scientists were establishing the scientific foundations of our modern understanding of these pediatric orthopaedic conditions.

## Introduction and background

Mary Cassatt (1844-1926) was an American fin de siècle artist who was unique in many ways. She painted and successfully exhibited her works when the art world was almost exclusively a male domain. She grew up in the United States but chose to live most of her life in Paris. Her paintings focused mainly on routine domestic scenes, and her subjects were almost exclusively females, many of whom were mothers, infants, and young girls [1,2].

Recently, Woodworth and Florescu discussed the relevance of Cassatt's painting Anne and her Nurse to human relations and to medical practice. They pointed out that, like Cassatt's unfinished canvas, human relations are always works in progress. They noted that the painting is a metaphor for the unfinished nature of clinical medicine, with its chronic, incurable illnesses and with all the uncertainties and stresses placed on physicians engaged in clinical practice [3].

The purpose of this review is to show how Cassatt's paintings relate to three pediatric orthopaedic conditions that were common 125 years ago and remain so now, namely, tibial torsion, metatarsus adductus, and asymmetric medial thigh skin folds.

## Review

Mary Cassatt was born in Allegheny City, Pennsylvania, and she grew up in Philadelphia where her father was a successful businessman. Her mother came from a wealthy banking family, and Cassatt enjoyed a nurturing and comfortable childhood. She traveled widely as an adolescent and was able to study at the prestigious Pennsylvania Academy of Fine Arts during the tumultuous years of the American Civil War. In 1865, she and her mother moved to Paris so she could attend art classes and hone her skills as a portrait painter. Her father, at first, did not appreciate nor approve of her career choice as an artist, but he nonetheless did support her financially, so she was able to continue to learn, to paint, and to associate with the Parisian avant-garde before returning to Philadelphia in 1870 at the outbreak of the Franco-Prussian War. In 1874, Cassatt moved back to Paris with her mother to resume her studies, and in 1877, she met Edgar Degas and began an association with the Impressionists that defined her artistic style for the rest of her career.

The term impressionism was at first meant to be pejorative. The influential French art critic Louis Leroy wrote a scathing review in 1874 of Claude Monet's painting Impression, soleil levant. Leroy described Monet's painting as looking more like an impression of something rather than a completed painting. Leroy clearly meant it as an affront to Monet and his colleagues. However, they gleefully adopted the term and referred to themselves as Impressionists, thus not only getting the last laugh but defining a new artistic genre as distinct from the prevailing Salon. We now recognize Impressionism as the art technique in which the artist uses color, natural light, and short brushstrokes to convey the message and meaning of the painting. Another art critic, Gustave Geffroy, described Cassatt, Berthe Morisot, and Marie Bracquemond as the three great ladies of Impressionism.

Tibial torsion

The ankle and foot of a child with internal tibial torsion (ITT) appear rotated or twisted internally relative to the axis of the thigh and the knee [4]. When the knee is flexed to 90 degrees and the thigh is in neutral rotation, the foot points inward to a varying degree, and the lateral malleolus is rotated forward and is more prominent. In 1903, le Damany published the results of his tropometric studies on the natural history of ITT (tropometer is the measurement of the angle between two planes or axes, in this case, the proximal and distal tibia). He showed that, in neonates, the axis of the proximal tibia and that of the distal tibia are essentially parallel and, in adults, the distal tibia is externally rotated ~20 degrees relative to the axis of the proximal tibia [5]. These numbers have generally been accepted by the medical community as the norm. However, Davids and Davis pointed out that "The anatomical definition of ITT is not precise and there is poor consensus concerning the optimal technique for its clinical assessment" [6].

Many clinicians find that defining ITT is somewhat like Justice Potter Stewart's attempt to define hard-core pornography. When opining in 1964 in the case of Jacobellis v. Ohio concerning the definition of hard-core pornography, Stewart said, in essence about hard-core, I cannot define it, but I know it when I see it. That pretty much sums up the situation with tibial torsion. We can quibble about the definition, but we know it when we see it, and so do parents and grandparents, and they often worry about it!

**Figure 1:**
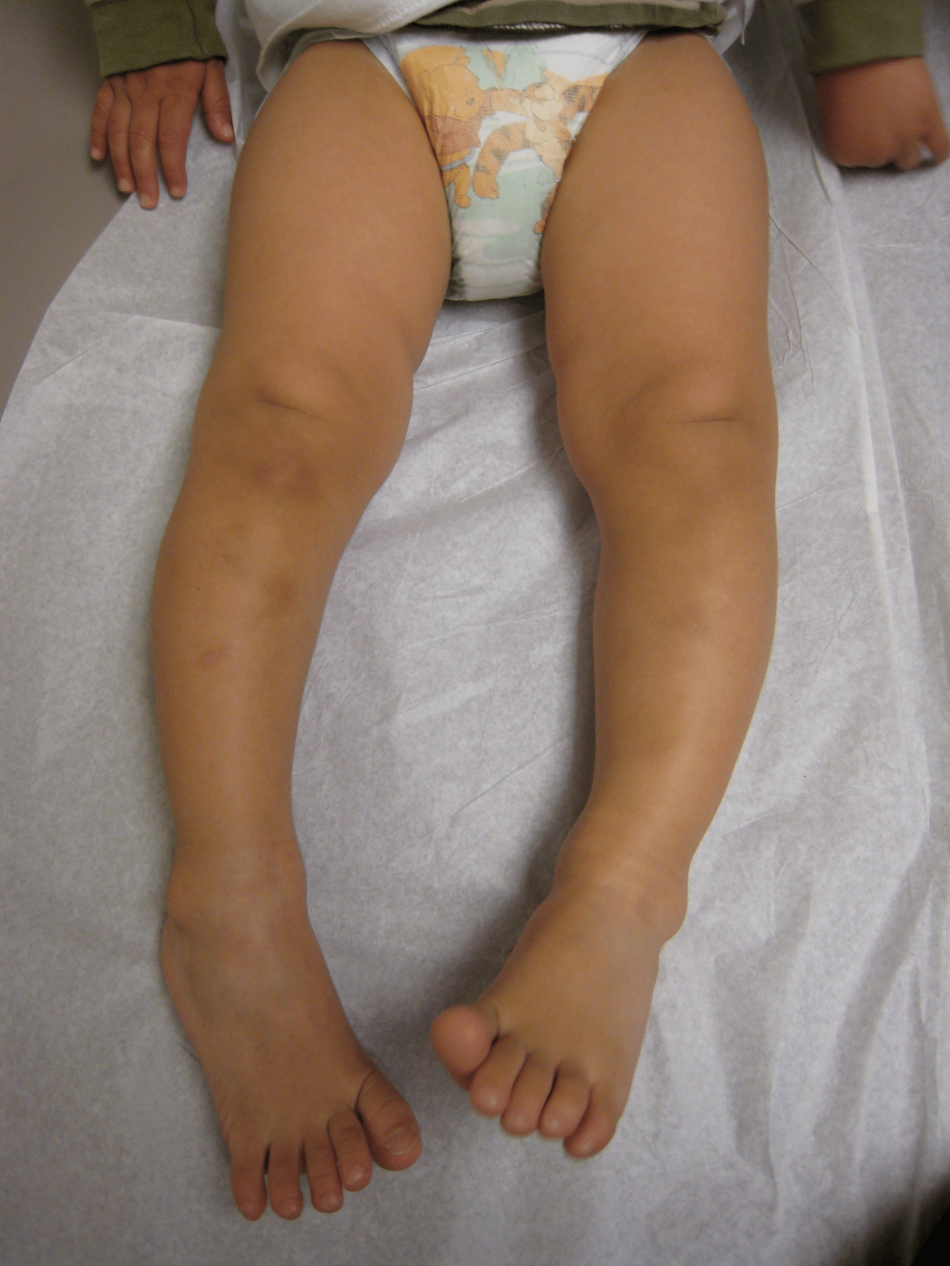
Apparent bowing of the legs This 14-month-old girl was referred because of bowing of the legs, and it does appear that the borders of the extremities are curved. When she walked, her knees appeared bowed, and the foot progression angle was 15 degrees internal. Image Credit: James G. Gamble

**Figure 2:**
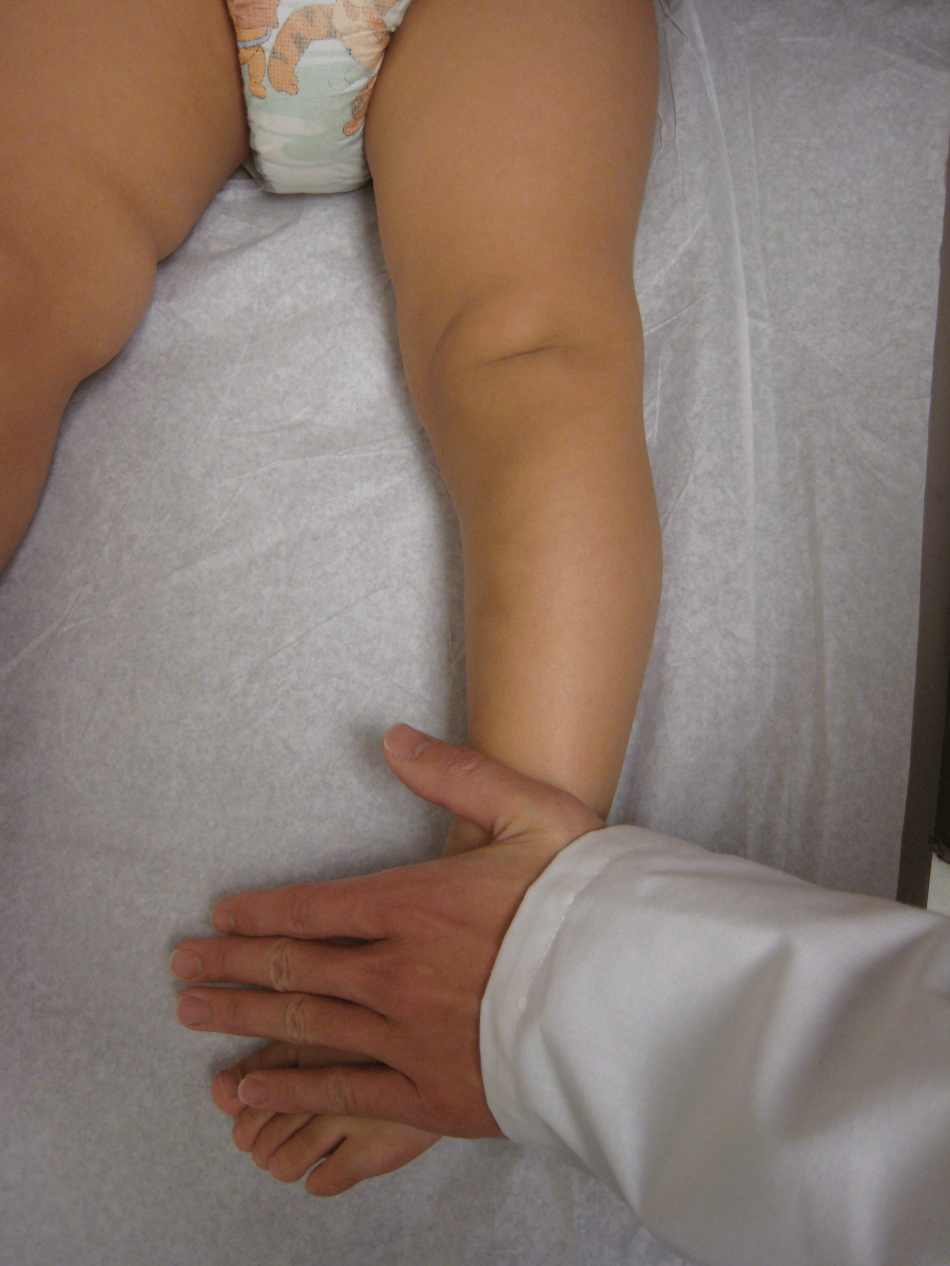
The cover-up test for this toddler The examiner places the knee in the neutral position with the patella straight up and covers the ankle and foot with his hand. Now, the thigh and proximal tibia appear straight and normally aligned, thus ruling out bowing of the knees. However, the toes point inward, so the cause of the deformity is distal to the knee and is due to the distal rotation of the tibia, i.e., tibial torsion. Image Credit: James G. Gamble

The incidence of ITT among infants and toddlers is high. It is often confused with bowing of the legs. A good way to tell the difference between bowing of the legs and tibial torsion is to use the cover-up test (Figures 1-2).

When children with ITT walk, they tend to in-toe relative to the line of progression, and they also frequently trip and fall. This tripping and falling is often the most disconcerting aspect for the parents who are concerned about potential injury and often point out black and blue spots on the legs of the toddler. Of course, all toddlers trip and fall frequently; that is part of being a toddler. 

**Figure 3:**
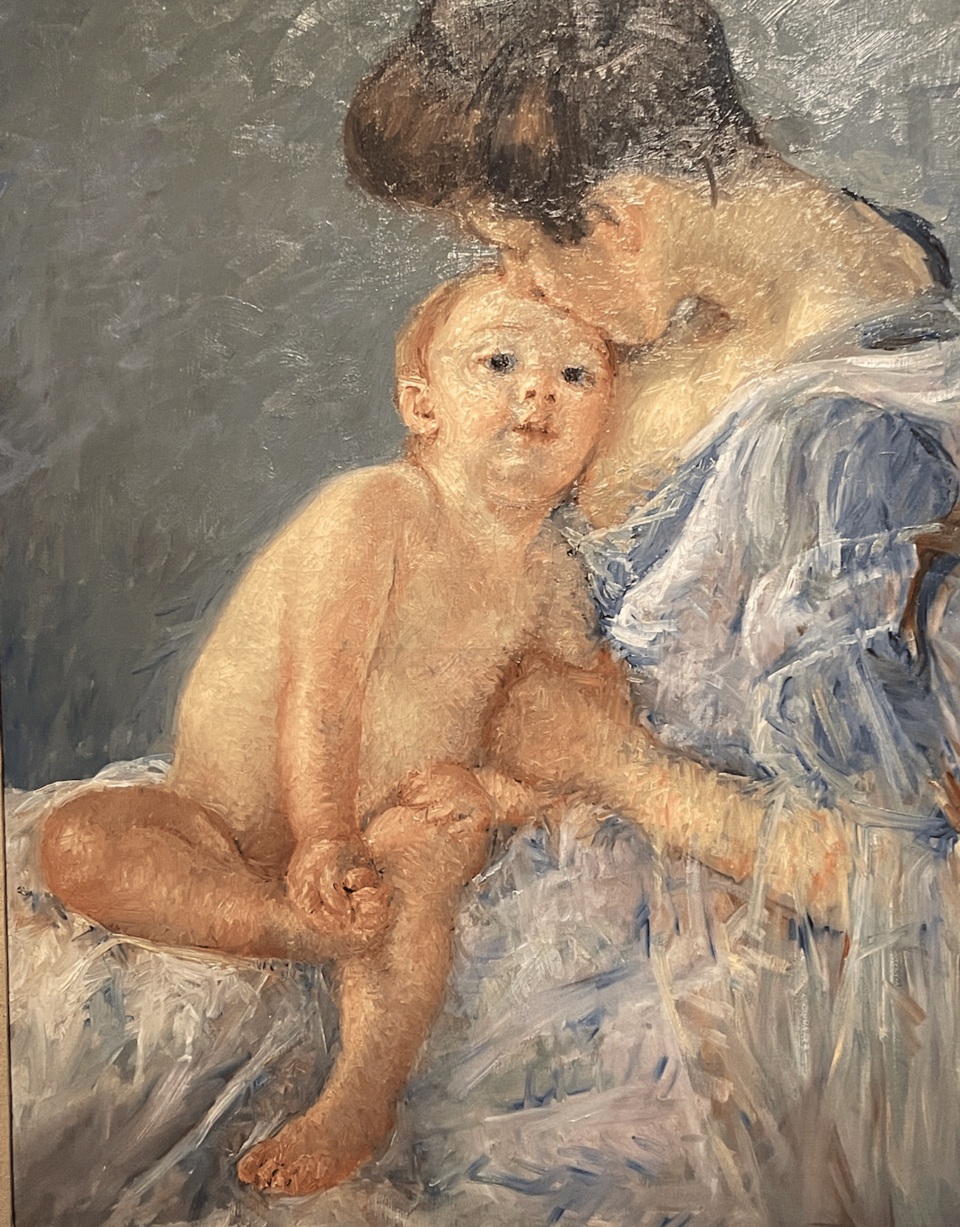
Maternity (Mother Kissing Her Baby), 1906 In this painting, the child's left knee directly faces the viewer. An imaginary line drawn down the long axis of the leg falls past the fifth toe. Cassatt's use of short brush strokes, light, and color give the clear impression of internal tibial torsion. Image Credit: From the personal photographic teaching files of James G. Gamble (permission for photography obtained from the San Francisco Palace of Fine Arts)

**Figure 4:**
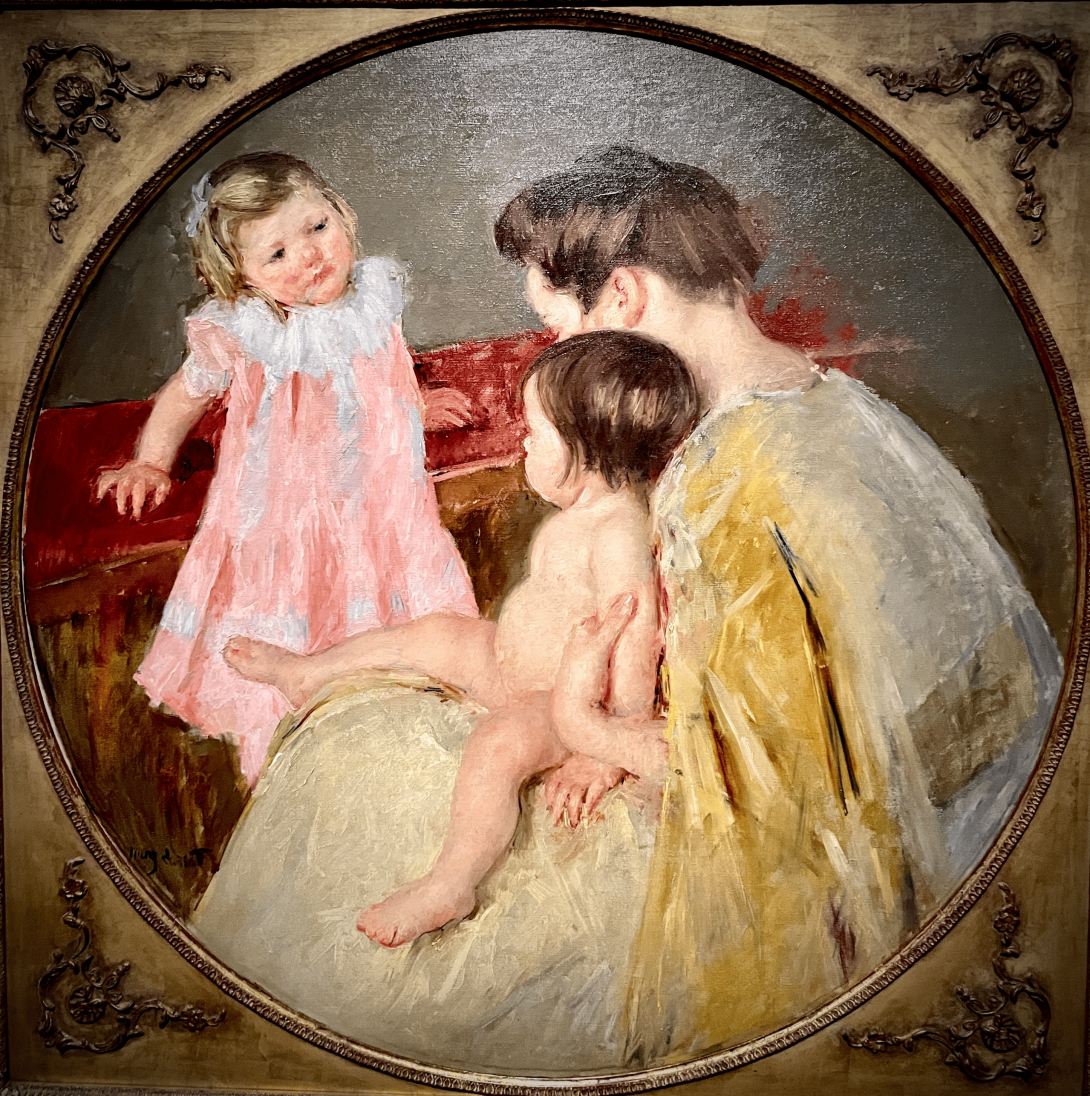
Mother and Two Children, 1905 An infant, most likely less than a year old, sits comfortably on her mother's lap with her knee flexed and her left foot internally rotated relative to the thigh and the knee due to left internal tibial torsion. Image Credit: From the personal photographic teaching files of James G. Gamble (permission for photography obtained from the San Francisco Palace of Fine Arts)

When a child with tibial torsion sits or reclines, the ankle and foot appear to internally rotate relative to the knee, and the lateral malleolus of the distal fibula is visibly prominent anteriorly. Cassatt captured this situation in some of her paintings (Figures 3-4).

In general, the natural history of tibial torsion for most children is spontaneous resolution with growth and development. Treatment, if any, should focus on education of the parents about the condition, provision of reassurance, and the offering of follow-up observation if needed.

Metatarsus adductus

**Figure 5:**
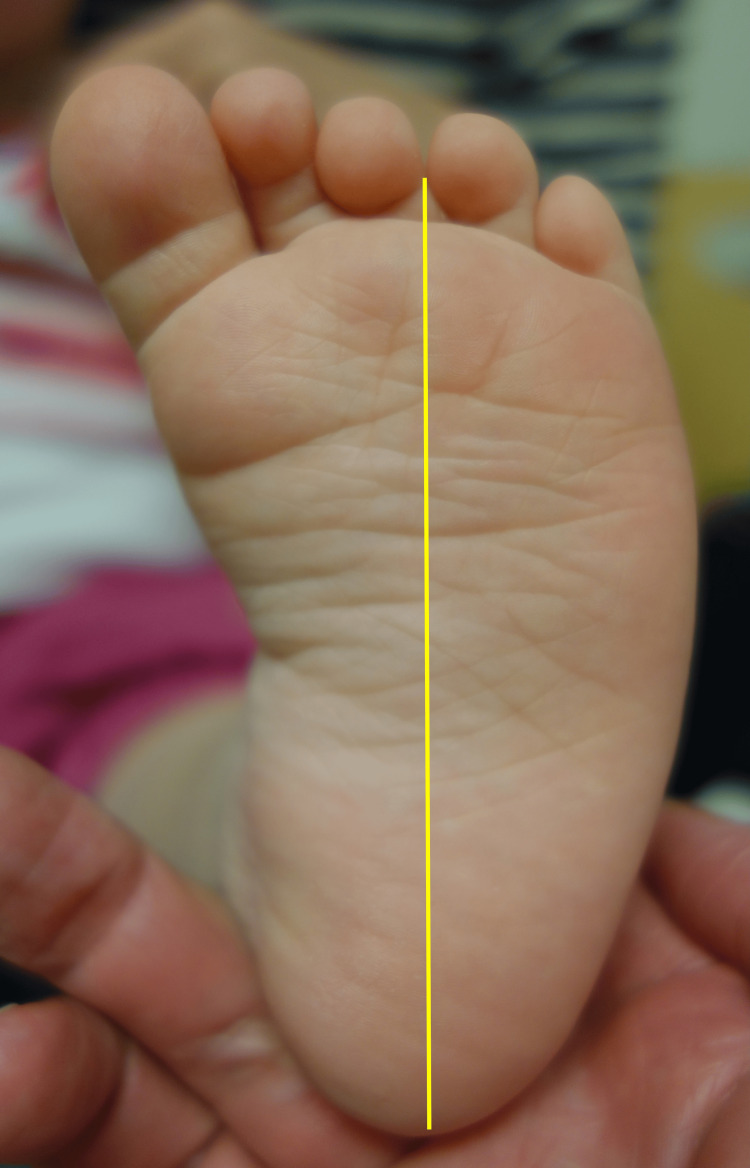
Plantar view of the left foot of an 11-month-old girl In children who have metatarsus adductus, the foot is shaped like a kidney bean, the lateral border of the foot is convex, the medial border is concave, and the heel bisector goes past the third toe. Image Credit: James G. Gamble

Metatarsus adductus is a foot posture in which the forefoot and the toes point inward relative to the hindfoot. The lateral border of the foot is curved and gives the foot a bean-shaped appearance. The heel bisector goes past the third toe (Figure 5). This same posture is apparent in several of Cassatt's paintings of infants and toddlers.

Like ITT, metatarsus adductus is common, and most children with the condition usually resolve spontaneously; occasionally, some may require a brief course of corrective casting.

**Figure 6:**
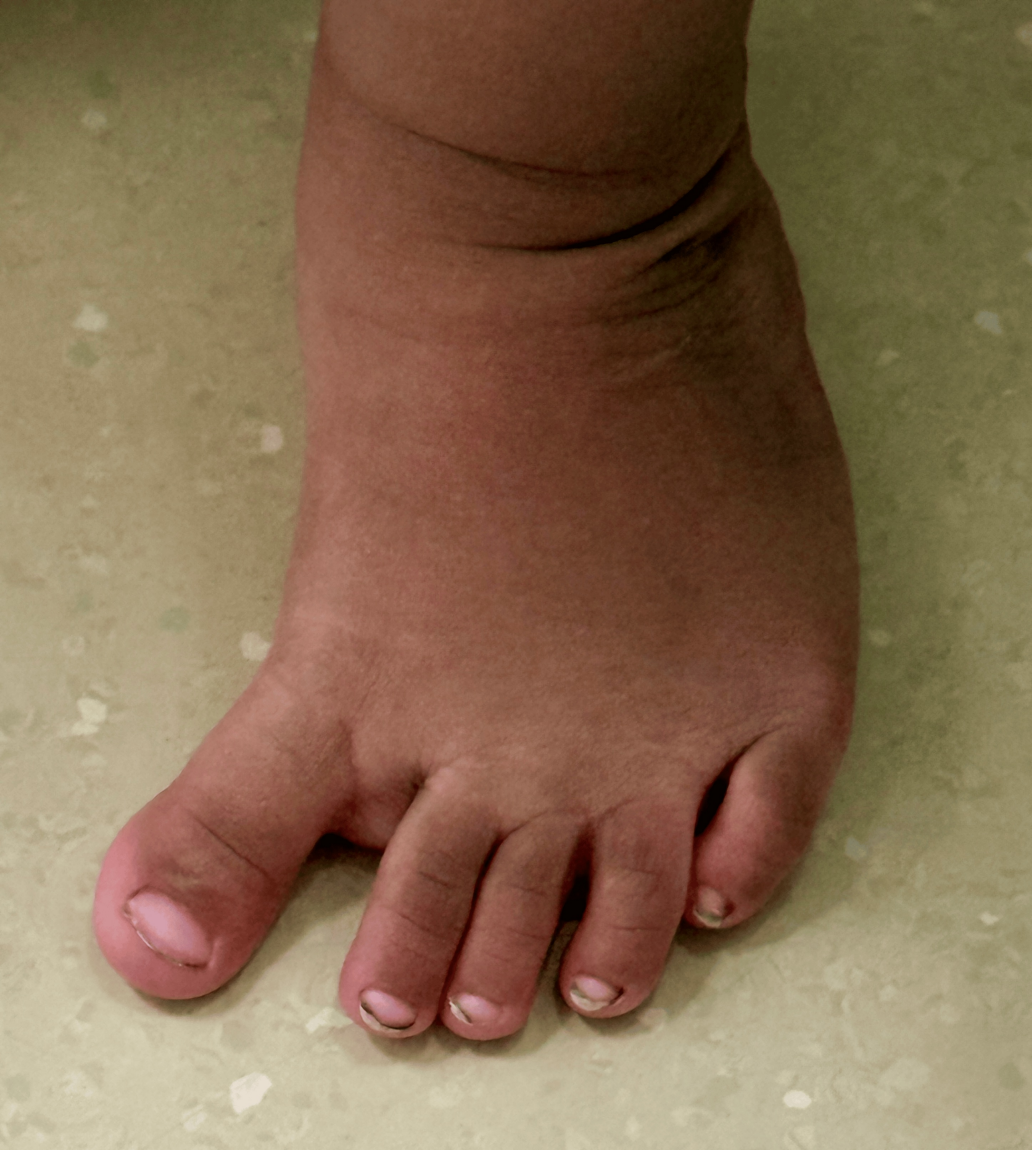
This 22-month-old toddler was referred for in-toeing This girl had a neutral thigh foot axis and transmalleolar axis. The heel bisector went to the fourth toe, but the foot was easily manipulated to the neutral position. When she walked, the great toe abducted, that is, "searched" medially, causing the in-toeing gait. Image Credit: James G. Gamble

A few infants and toddlers have a dynamic internal rotation of the foot and leg due to a combination of metatarsus adductus and an overactive adductor hallucis muscle. The lateral border of the foot is more C-shaped than in metatarsus adductus, the toes point inward, and the most remarkable feature is that the first interdigital space widens as the child takes a step (Figure 6). The foot can be corrected easily back to the neutral position with passive stretching, but it bounces right back if the foot is released. When these children walk, the foot appears to have a searching great toe, that is, a dynamic hallux varus that pulls the foot inward, increasing the interdigital distance and making the great toe and foot look like a prehensile appendage, ready to grasp anything.

**Figure 7:**
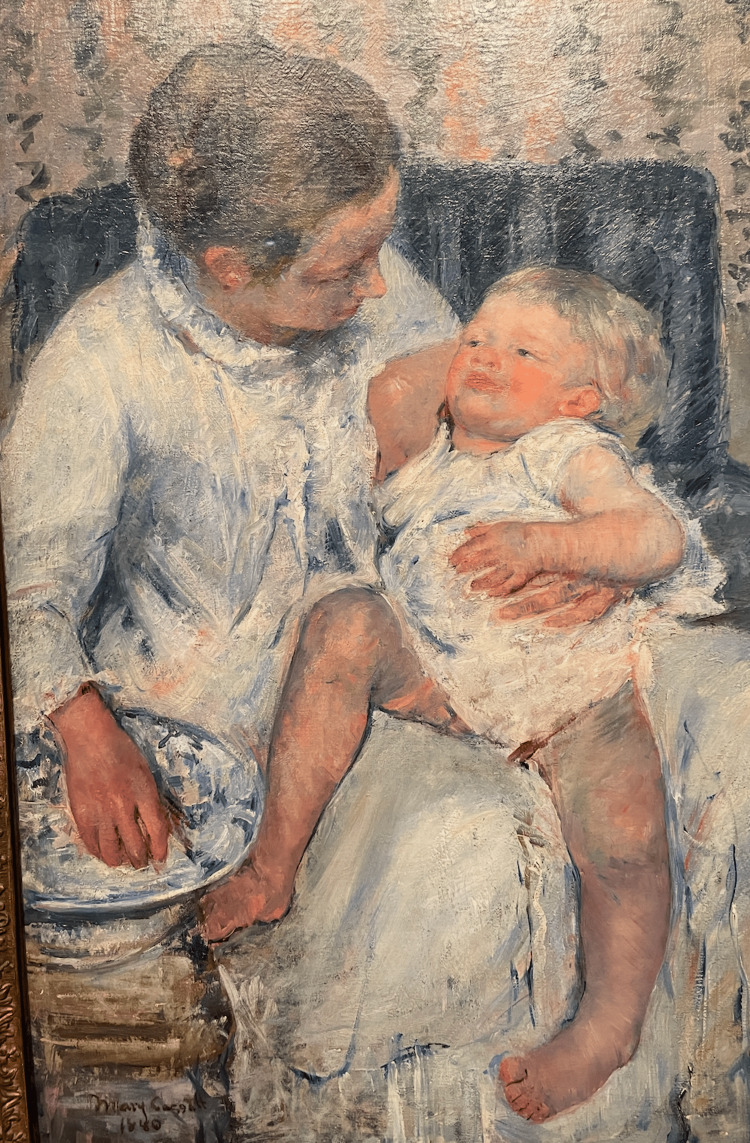
Mother About to Wash Her Sleepy Child, 1880 The baby's left leg is internally rotated relative to the thigh, and the foot is internally rotated and supinated. Image Credit: From the personal photographic teaching files of James G. Gamble (permission for photography obtained from the San Francisco Palace of Fine Arts)

In Figure 7, Cassatt painted a child with an internally rotated left leg due to ITT. Also, the foot is supinated, and the great toe is extended and adducted. It is easy to imagine this child having a dynamic adduction and supination of the foot with a searching great toe.

Although most children with tibial torsion and in-toeing gait resolve with growth and development [7,8], children with clubfoot or those with neuromuscular disorders do not always resolve and may require orthotic or surgical intervention.

Asymmetric thigh skin folds

Hip dysplasia and dislocation, also known as developmental dysplasia of the hip (DDH), occurs less frequently than tibial torsion and metatarsus adductus, but if untreated, it has much more severe, life-lasting consequences. Untreated DDH can result in a limp, disability, hip arthritis, and chronic pain. Early diagnosis is crucial to achieving a good outcome, and it is particularly important to diagnose the condition before the child begins to walk. Physical examination signs such as the positive Ortolani and Barlow usually disappear by the time the child is 4-6 months old. Other subtle signs, such as limited hip abduction and asymmetric skin folds, become more obvious.

**Figure 8:**
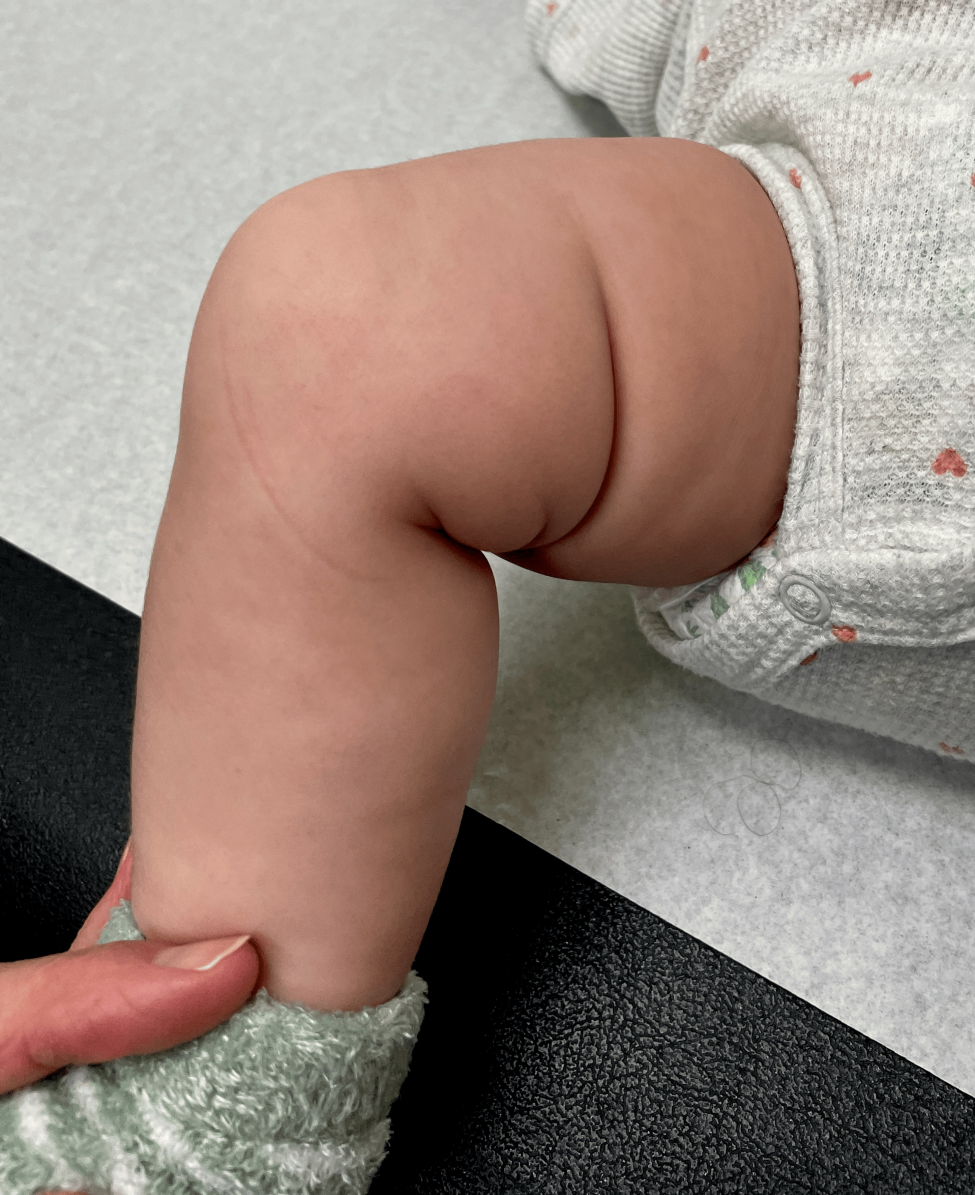
This 15-month-old girl was referred for a limp and a short limb This girl has an asymmetric medial thigh skin fold and a radiographically documented right hip dislocation. Image Credit: James G. Gamble

The extremity of a child with DDH often appears shorter than the contralateral side due to a proximal migration of the entire extremity as the femoral head no longer resides within the confines of the acetabulum. This shortening causes soft tissue changes [9] such as a deep medial thigh skin fold (Figure 8) that is not present on the uninvolved side.

Pediatric orthopaedic textbooks identify asymmetric thigh skin folds as an important physical sign of DDH [10,11]. However, the specificity and sensitivity of this sign have been reported to be low [12,13] because some children without DDH can have asymmetric thigh skin folds.

**Figure 9:**
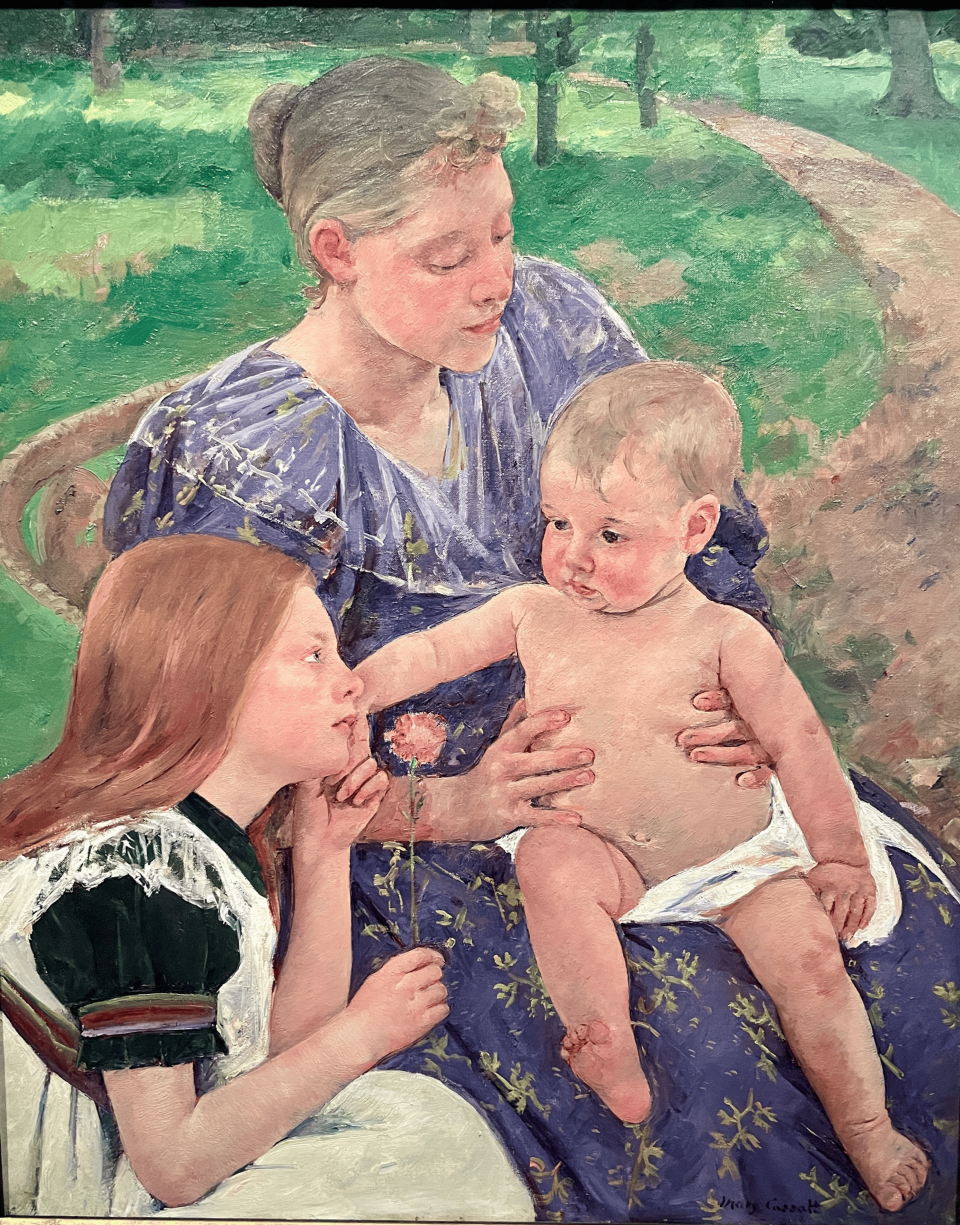
The Family, 1892 Cassatt's painting depicts a mother and sister intently focused on a robust infant with a flexed and externally rotated right hip and a deep medial thigh skin fold. Image Credit: From the personal photographic teaching files of James G. Gamble (permission for photography obtained from the San Francisco Palace of Fine Arts)

In The Family, 1892 (Figure 9), Cassatt painted an infant with a right-sided deep medial thigh skin fold that is not present on the left side. It is highly unlikely that this amount of asymmetry would be due simply to the position of the limb. A deep medial thigh skin fold like this does not form only with hip flexion, abduction, and external rotation, thus greatly increasing the suspicion for DDH.

Fortunately, the incidence of DDH has decreased since Cassatt's time due, in part, to a better understanding of the pathophysiology and modern noninvasive imaging techniques such as ultrasound [10]. However, just as important has been our attempts to convince people to abandon the custom of swaddling infants that was almost universal during Cassatt's time.

Any attempt to make a medical diagnosis from paintings has inherent difficulties. Two major limitations are confirmational bias and the retrospective nature of the process. Furthermore, we have no clinical information about the subjects, and the information available reflects a static posture which may misrepresent reality.

## Conclusions

Like Frida Kahlo, but to a somewhat lesser extent, Mary Cassatt's reputation and interest in her work have greatly increased. Museums such as the Metropolitan Museum of Art in New York and the Museum of Fine Arts in Boston prominently display her paintings. The exhibition Mary Cassatt at Work, organized by the Philadelphia Museum of Art, was a huge success in the United States. In October 2025, her painting Sara, with Bonnet Streamers Loose, Feeding Her Dog sold at Phillips Auction for $914,400. Other paintings have also fetched six figures. The United States Postal Service recognized her importance and released a 23-cent stamp with her portrait as part of their Great Americans series. They also released five other lower-denomination stamps featuring her paintings.

Cassatt loved Paris and thrived in the fin de siècle artistic community. Her brother, Alexander, was independently wealthy as president of the Pennsylvania Railroad. He and Mary collected many important works of the Impressionists. Towards the end of her life, Cassett suffered with diabetes, rheumatism, neuralgia, and cataracts, undergoing four unsuccessful ophthalmologic operations. Loss of her eyesight was devastating and made painting impossible. She died at her Château de Beaufresne outside of Paris in 1926 at the age of 82. It is an interesting coincidence of time and place during the fin de siècle that Cassatt was in Paris painting children with common pediatric orthopaedic conditions like ITT and metatarsus adductus, while Pierre Germain Marie le Damany was in Rennes scientifically documenting the natural history of tibial torsion, and Wilhelm Conrad Röntgen was across the border in Würzburg having been awarded the first Nobel Prize in Physics for his discovery of X-rays that would forever revolutionize the way we diagnose and treat orthopaedic conditions.
